# Tech Ethics Through Trust Auditing

**DOI:** 10.1007/s11948-022-00379-0

**Published:** 2022-06-07

**Authors:** Matthew Grellette

**Affiliations:** Institute on Ethics and Policy for Innovation, McMaster University, Hamilton, Canada

**Keywords:** Trust, Public participation, Tech ethics, Virtue by design, Emerging technology, STS

## Abstract

The public’s trust in the technology sector is waning and, in response, technology companies and state governments have started to champion “tech ethics”. That is, they have pledged to design, develop, distribute, and employ new technologies in an ethical manner. In this paper, I observe that tech ethics is already subject to a widespread pathology in that technology companies, the primary executors of tech ethics, are incentivized to pursue it half-heartedly or even disingenuously. Next, I highlight two emerging strategies which might be used to combat this problem, but argue that both are subject to practical limitations. In response, I suggest an additional way of augmenting the practice of tech ethics. This is to employ “trust audits,” a new form of public participation in the socio-technical environment. In the remainder of the paper, I offer a description of how trust audits work, what they might look like in practice, and how they can fit in alongside those other strategies for improving tech ethics.

## Introduction

In recent years, many technology developers have embraced a so-called “wrecking ball ethics,” centered on norms such as “move fast and break things” and “build it first and ask for forgiveness later” (Taneja, 2019; Singer, 2018; Powles & Véliz, 2016). In doing so, they have eschewed accepted institutional norms and regulatory pathways as hurdles to innovative efficiency and creativity. Yet, many of these rules and principles were specifically designed to slow down the process of technological innovation for a very good reason: the protection of the public. Thus, bypassing them has predictably led to numerous corporate scandals and problematic expressions of state power (Metcalf & Moss, 2019).

As a result, recent polls show that the public’s trust in the technology sector is crumbling. Pew Research, for example, reports that the number of American with a “positive view” of technology has tumbled 21 percentage points over the last four years, from 71 to 50% (Doherty & Kiley, 2019). Similarly, the 2020 Edelman Trust Barometer reports that 61% of respondents to its global poll believe that "technology is out of control” (Edelman, 2020). These results are worrisome for technology developers and for the nation-states which support and rely upon them because, without the public’s embrace, many new technologies will effectively be stillborn (European Commission, 2020). Thus, with an eye to soothing the public’s discomfort, many technology companies and governments have started to publicize a commitment to “tech ethics”. That is, they have pledged to design, develop, distribute, and employ new technologies in an ethical manner (Munro, 2019; Tepper, 2020).

Understood as such, tech ethics is not merely an academic or intellectual activity, it is the practice of participating in the technology sector in a morally acceptable way. As desirable as this sounds, I will suggest that there is good reason to doubt whether tech ethics, as it is currently instantiated, is likely to increase public trust in technology developers. In particular, I will explain how the practice of tech ethics is currently subject to a pathology that is actually exacerbating the public’s growing distrust of technology developers. I will then identify two existing suggestions as to how the practice of tech ethics might be improved, before noting key problems with each of those approaches. Finally, I will identify and briefly describe an alternative strategy for augmenting the practice of tech ethics.

## A Standing Problem

If technology developers really were to commit themselves to design, develop, distribute, and employ new technologies in an ethical manner, this would likely go a long way towards bolstering public trust in the technology sector. However, as things stand now, there is reason to be cynical about this possibility. Specifically, most technology developers are currently incentivized and empowered to pursue tech ethics half-heartedly or even disingenuously.

In today’s world, the bulk of new technologies are created by private businesses rather than by governments or non-profit institutions (Rodrik, 2020). The result is that most technology developers are preoccupied by an overriding concern with generating revenue. Every technology company needs to create enough income to sustain its operations. Plus, many such businesses go further and embrace a “shareholder approach” towards their operations, thereby committing to always act in ways that will maximize their profits (Danielson Heck & Shaffer, 2008). At the same time, these companies tend only to be governed with a regulatory “light touch”, which affords them significant operational discretion (Australia, 2014; Watts, 2019).

All this creates a problem for tech ethics because choosing to act ethically can come with significant financial costs. First, ethically oriented companies may have to refrain from certain revenue-generating activities. In a 2008 report, for example, the Special Representative of the United Nations Secretary-General noted that ethical businesses must take precautions against being complicit in human rights abuses (Ruggie, 2008). Ethically oriented companies are therefore precluded from making use of many inexpensive production facilities around the world and from working with or selling to a number of other businesses and governments. Second, ethical companies may be required to take certain costly actions that an unethical competitor would not. For example, it has long been suggested that ethically run businesses should engage in regular and extensive stakeholder consultations, even when this would not help to advance their market position (Langtry, 1994).

The point here is that the costs associated with acting ethically can impinge on a technology company’s revenues, even to the point of threatening its continued existence. As such, there is a clear business rationale for technology companies to limit their commitment to tech ethics when doing so threatens to significantly undercut their profits (Duska, 2000). This tension between ethics and profits is even more pronounced when businesses operate according to the shareholder approach. A company committed to maximizing its profits will be averse to *any* ethical action that would infringe on its bottom line. So, when shareholder-focused companies purport to embrace the practice of tech ethics, their commitment is only to do so if and when it will ultimately make them money.

The fact that technology companies are both motivated and free to chase profits means that many of them will only ever be weakly committed to the practice of tech ethics. But things are worse than this. As noted in the introduction, both businesses and governments view tech ethics as a means to combat public antipathy towards developers and their products. From a business perspective, the cost of embracing tech ethics is therefore viewed as an investment whose return will be the creation of enough public trust to facilitate the eventual sale and use of a company’s products and services. The problem is that instrumentalizing tech ethics in this way can lead businesses to some rather unpalatable conclusions about how to engage in that practice. Specifically, if tech ethics is viewed simply as a means of building public trust, then what should a business do if it were possible to create such trust by erecting a mere façade of participation in tech ethics? According to the logic of investment, if one can receive the same return on either of two differently priced options then—all other things being equal—one should invest in the cheaper alternative. Hence, if it were possible for a company to reap the rewards of appearing to behave ethically without having to pay the costs associated with actually doing so, then this is what they should do. To be clear, this is no mere hypothetical. Over the last few years, numerous technology companies have been caught paying mere lip service to the norms of tech ethics and implementing ethics processes that were designed to be ineffectual showpieces (Hao, 2019).

Thanks to the significant media attention that these discoveries have generated, the public is now keenly aware that technology companies are subject to these problematic incentive structures and that they cannot, therefore, be trusted to think and act ethically, even when they claim to be doing just that. The result is a situation that is so obviously counterproductive to the task of restoring public faith in technology developers that we must ask whether anything can be done to improve it.

## A Pair of Corrective Strategies

Some have suggested that the best way forward is to take final responsibility for tech ethics out of the hands of businesses and give it to outside agencies such as state actors or international organizations. More specifically, the thought is that since technology companies are abusing the operational freedom that comes with a light regulatory touch, perhaps it is time for countries to adopt a more robust regulatory approach—one that would directly compel these businesses to design, develop, distribute, and employ new technologies in conformance with the demands of morality (McNamee, 2020; Radu, 2020). This strategy has already been widely promoted within the European Union and is steadily gaining popularity in other parts of the world (O’Brien, 2020; European Commission, 2020; European Union, 2020). Even technology entrepreneurs and technology companies have started to call for the creation of top-down regulatory frameworks to govern the development and use of ethically fraught innovations such as facial recognition tools (Herrera, 2020; Levinson-King, 2019).

Yet, as popular and straightforward as a robust regulatory approach seems to be, there are a number of reasons to question how significantly it would advance the practice of tech ethics. First, some scholars have pointed out that the content of regulatory schemes inevitably lag behind the ethical issues raised by the newest technological developments. This issue, known as “the pacing problem,” comes down to the fact that "technology changes exponentially, but social, economic, and legal systems change incrementally" (Downes, 2009). By the time a regulatory system has been designed and enacted to govern over the technological status quo, a new set of technologies will have emerged, bringing with them a novel set of ethical issues that the existing framework handles awkwardly, vaguely, or not at all (Tricoles, 2019).

A second concern with developing more robust regulatory regimes is that the agencies likely to create and impose such frameworks would be just as susceptible to half-heartedly or disingenuously instantiating ethical governance as the businesses that they are supposed to oversee. The agencies most likely to be put in charge of regulating tech companies are state or regional governments, since they are often the best resourced and are possessed of the requisite political authority. But while their political rhetoric is generally laden with moral language, state governments are often willing to engage in illicit activities as a means of advancing national or regional interests. Hence, putting these institutions in a position of regulatory influence may just create opportunities for state governments to compel tech companies to serve those countries’ own unethical ends. This issue is well-illustrated by the fact that a number of different countries have already established laws mandating that digital tech companies be required to share users’ personal data with security authorities and to include “hidden censorship and surveillance functionalities” in their products (Flyverbom Delbert & Matten, 2019).

A third issue is that not every part of the world plays host to state or regional governments that are sufficiently well-resourced to effectively impose the demands of tech ethics on the companies operating therein. To the contrary, a pair of recent reports note that many low-income governments have such a “limited capacity and insufficient resources” that they are incapable of carrying out even critical regulatory functions such as realizing basic safety assessments of new technologies (Kaddu D’Amore Clark & Nkansah, 2018; Pombo Porrás Saido & Cascio, 2016). The idea of using regulatory systems as a panacea for the practice of tech ethics is, therefore, a conceit of wealthier countries and regions.

Given these worries about the regulatory approach, some have searched for another way to enhance the practice of tech ethics. One idea that is quickly gaining adherents is to build ethical assessments and review procedures right into the design and development phase of each new technology (Friedman & Hendry, 2019; Winkler & Spiekermann, 2018; Tatum, 2004). Some, for example, have suggested that the designers of digital technologies should seek to identify any negative impacts that new interfaces can have on users’ psychological well-being and adjust their products to minimize any such harms (Peters Calvo & Ryan, 2018). The ostensible power of this “design approach” is that it would limit tech companies’ ability to act unethically, since they would be forced to create new products in a way that is sensitive and responsive to matters of ethical concern. At the same time, it avoids issues like the pacing problem, since it frontloads the practice of ethics into the innovation process, thereby allowing us to identify and resolve any problems with a product before it is ever manufactured or distributed (Manders-Huits, 2011).

Yet, despite these and other potential advantages, there is reason to question whether the design approach can actually overcome the current problem with tech ethics. Proponents of this strategy often envision it playing out within the work of “researchers, designers and engineers” who are so well trained and conditioned in the practice of ethics that they will subject their own work to ethical scrutiny and then flag, adjust, or even abandon their projects if it is called for by their ethical findings (Cummings, 2006; Friedman & Hendry, 2019; Peters, 2019). However, the fact that tech companies are subject to the profit-motive means that most of them are uninterested in authorizing, let alone encouraging, their researchers, designers and engineers to engage in this potentially costly form of decision-making. To the contrary, most technology companies employ an incentive structure that is focused on prompting research and development professionals to focus their efforts solely on the efficient launch of new and innovative projects. Thus, as Roel Dobbe and Morgan Aimes note, such employees might enter into the workforce having been educated in ethics, but the conditions of their employment will often dissuade them from “meaningfully and proactively addressing the social implications of new technologies” (2019).

With this problem in mind, some proponents of the design approach have suggested that corporate decision-making structures need to be reimagined and reconfigured so that designers and engineers are afforded greater ethical influence over their companies’ technological activities (Dobbe & Ames, 2019). But, again, given their existential focus on generating profits, it is unclear whether many technology companies would be interested in making these sorts of adjustments. Thus, there is good reason to question whether the design approach will ever be so widely embraced by such businesses as to be capable of renewing public faith in the technology sector.

## A Different Strategy

To sum up the discussion so far, while technology companies often have final responsibility for the implementation of tech ethics, they are disposed to be unreliable in fulfilling that role. Moreover, the most popular strategies for overcoming this problem appear to be subject to some significant limitations. A robust regulatory scheme is unlikely to fully remedy the situation thanks to issues such as the pacing problem, unethical state agendas and, in many parts of the world, a sheer lack of resources. Moreover, it is questionable whether technology companies will be willing to adjust their decision-making structures in the way that seems to be necessitated by the design approach. Challenging as these issues are, they may not be ruinous for tech ethics. There may, after all, be other ways to advance the quality of this practice aside from the regulatory and design approaches. In what follows, I will propose one such alternative, which is that technology developers might be pressured into taking tech ethics more seriously through the use of “trust audits,” a novel form of public participation in the socio-technical environment.

To understand this suggestion, it is necessary to briefly consider the nature of trust and trustworthiness. There is, admittedly, philosophical disagreement about some of the more esoteric aspects of trust (McLeod, 2020). However, there is also significant consensus concerning its basic elements. In particular, most will agree that an instance of trust involves one agent (a trustor) believing that another agent (a trustee) can be relied upon to act in some way that the trustor would desire—even when the trustee does not otherwise have to act in that way (Baier, 1986; Hardin, 2002; McLeod, 2020). Thus, few will deny that I trust my dog-walker to do her job insofar as I believe that she will come by the house and take my dog for a walk a few times every week, despite knowing that she could choose not to show up.

A couple of “clear conditions” for judging someone’s trustworthiness follow from this rudimentary understanding of trust. Namely, we must consider whether a potential trustee is both “competent and willing to do what they are trusted to do” (McLeod, 2020). Now, to say that there is good reason to believe that an agent is *willing* to act in a certain way just means that one is justified in thinking the agent will be motivated to perform an action. For example, I may have good reason to think that my dog-walker is adequately motivated to walk my dog because I know she is a possessed of an admirable moral character (Simpson, 2013) and/or that she has an interest in receiving money that I am offering for a job well done (Hardin, 2002). On the other hand, to say that there is good reason to believe that someone is *competent* to perform an action means that one is justified in thinking that they are possessed of the knowledge, skills, and resources needed to do so (Hardin, 2002). For instance, I have good reason to think that my dog walker is competent to walk my dog because I know that she is strong and skilled enough to handle him, that she has set aside time to walk him, that she has access to my apartment, etc.

This is all relevant to the issue of tech ethics because, just as it is possible to evaluate another person’s trustworthiness by assessing their motivations and competencies, so too can one evaluate the trustworthiness of institutions like technology companies (Govier, 1997; Ryan, 2020). And insofar as this is the case, it should be possible to assess whether such entities should be thought trustworthy with respect to the practice of tech ethics. For example, learning about whether a given technology company is shareholder- or stakeholder- focused, the attitudes and activities of its employees, the character of its executives, and the nature of its governance structures will take one a long way towards discerning whether it is inclined to act ethically. One can also glean a significant amount about the ethical orientation of a company by considering the quality of its business practices, including the transparency and honesty of its communications and especially the reliability and safety of its products and services. At the same time, there are numerous considerations that might tell whether a technology company is competent to act ethically. For instance, a business that is on the verge of bankruptcy and whose products are only desired by despotic governments would not appear to have the fiscal resources necessary to withdraw from its unethical business dealings. Thus, all other things being equal, it can be judged incompetent to effectively practice tech ethics.

My proposal is just that the foregoing sorts of evaluations might be used to pressure technology companies into more fully embracing tech ethics. To see how this could work, consider that determinations of trustworthiness are a potent sort of practical judgment that can affect how people will choose to interact with other agents and agencies. In particular, positive judgements of trustworthiness are widely associated with a willingness to place oneself in a position of vulnerability to a trustee with regard to some act or activity (Baier, 1986; Ryan, 2020). For example, the judgement that my dog walker is trustworthy has led me to put myself in the vulnerable position of providing her with unsupervised access to my apartment and the opportunity to harm my best friend. In much the same way, positive judgments of a company’s ethical trustworthiness are correlated with a willingness to purchase its products and services, and even with developing a sense of brand loyalty (Gana & Koce, 2016; Nguyen Leclerc & Leblanc, 2013). Importantly, this effect also operates in the other direction. Negative judgments of an agent or agency’s trustworthiness disincline people from making themselves vulnerable to that entity. Moreover, if people find out that an entity which they already trust has been behaving in a way that violates their confidence, they are likely to experience feelings of betrayal and punitive anger towards their former trustee (Baier, 1986). Hence, negative judgments about a technology company’s ethical trustworthiness leads people away from purchasing or endorsing its products, let alone developing or maintaining any sense of brand loyalty towards it (Gana & Koce, 2016). In addition, should such a business be discovered to be violating the trust of its existing customers, it should expect to face criticism, shaming, boycotts, and other forms of public censure that would further hurt its bottom line.

These patterns of behaviour suggest that if technology companies were publicly evaluated for their ethical trustworthiness, they might be pressured into more fully embracing the practice of tech ethics. Imagine, for example, that a group of concerned citizens were to start producing and distributing reports meant to inform the public about the ethical trustworthiness of specific technology companies. It seems that the more influential these assessments—these “trust audits”—became in determining public opinion, the more pressure there would be on potential auditees to organize their operations so as to reap the benefits of being judged ethically trustworthy and to avoid the ramifications of being found untrustworthy. Moreover, the more prevalent these trust audits become, the more companies would have to view themselves as potential auditees and be incentivized to adjust their operations in order to receive favourable reports. It follows that if trust audits became prevalent and influential enough, they could conceivably prompt widespread improvements in technology companies’ ethical dispositions and competencies. Furthermore, if members of the public were to find themselves capable of targeting and influencing the operations of even a few technology companies in this way, their sense that technology is “out of control” would surely lessen. Instead, they would experience themselves wielding a form of communal or democratic power capable of pressuring technology companies into being more ethically trustworthy agencies.^1^

With all this in mind, there is reason to think that trust audits might constitute a form of public action that could both prompt individual technology companies into better alignment with the practice of tech ethics and help to regrow public trust in the technology industry.

## Envisioning the Practice

Given the potential that trust auditing appears to hold in the abstract, it is worth thinking about what such evaluations might actually look like in practice:

To begin, it seems reasonable to expect that individual community members and their leaders would be interested in seeing some version of trust auditing come to fruition. As noted in the introduction, public trust in technology developers is quickly and significantly declining. Thus, now more than ever, private citizens are positioned to understand the importance of using trust audits to distinguish ethically oriented enterprises from those less well-inclined. Similarly, community leaders are likely to view trust audits as a way of identifying technology companies that are truly disposed to respect the interests and needs of local individuals and to establish fair and equitable relationships with local institutions. Consequently, if promoted effectively, trust audits seem primed to garner public favour.

At the same time, it is important to recognize that trust audits would be a complex and somewhat resource-intensive undertaking. At the least, they require auditors to have the time and the means to identify, collect, and interpret significant amounts of technical information and to make complex inferences about a company’s ethical disposition and competencies. It would, therefore, be unrealistic to think that many private citizens would be interested, let alone capable, of performing their own trust audits. Instead, both community members and community leaders would likely want to identify or appoint a team of dedicated trust auditors whose work would be shared publicly. In this vein, some communities might delegate the job of trust auditing to local government, others might prefer to have trust audits be performed in association with a local university, while others might desire that these evaluations be undertaken by a panel of public volunteers.

In addition to being adequately resourced, trust audits must be viewed as credible sources of information by the populations they are meant to serve. Auditing teams must therefore be constituted in ways that accord with the epistemic and ethical norms of their communities. For example, because different communities view different social roles as embodying ethical authority, some may look to local religious figures to contribute, while others might prefer to involve professional ethicists, and still others might think that this is a role best suited to members of the legal profession or some other sort of recognized moral leader. At the same time, determining the disposition and competencies of complex organizations like technology companies is no easy feat, especially when these institutions may have a vested interest in disguising their real motivations or manipulating their public image. It is therefore essential for auditing teams to include subject-matter experts who can speak to the nature of the technologies that a company is producing as well as to the tenor of that company’s business practices. As such, auditing teams are likely to include some combination of academics, technology specialists, members of professional societies and trade unions, members of the local business community, and others with relevant expertise. Moreover, there is nothing to preclude auditing teams from consulting with outside experts, especially if this is deemed necessary to the production of an accurate assessment. The other major consideration, when it comes to credibility, is the danger of trust audits being unduly influenced by technology companies or other stakeholders. With this threat in mind, individual trust auditors will likely be vetted for potential conflicts of interest, and trust auditing teams will need to be resourced and managed in ways that ensure their analytic independence.

Once constituted, trust auditing teams will aim to produce assessments that are comprehensible to most members of the public and yet detailed enough to explain and justify the auditors’ conclusions to critical readers. As such, their work will be best formulated and promoted as independent panel reports, akin to those created by legal or governmental working groups. Such reports generally consist of detailed findings of fact and of the reasoning that the panelists used to reach their empirical and normative conclusions. They may even include the concurring or dissenting opinions of various panel members. These reports can be disseminated as both summary- and long-form documents, allowing members of the public to either quickly scan the auditors’ claims or to investigate the logic of their evaluations more fully.

Insofar as trust audits provide a detailed account of the evidence, the reasoning, and the conclusions of an independent body of local ethical and subject matter experts, they offer more detailed and authoritative judgments than everyday journalism. That said, trust audits will ideally be shared through social media and be reported on by local media outlets in whatever forms are most efficacious in that locality, be it television, newspaper, radio, etc. After all, the point and purpose of trust audits is to increase public awareness and responsiveness to the trustworthiness of various technology companies. But, more than this, the involvement of social and popular media offers auditees an opportunity to publicly respond or even to challenge trust auditors’ reasoning. For example, a company might contest an auditing teams’ framing of an issue or it might release countervailing information (Wynne, 2007). In this way, trust audits might actually prompt technology companies into public discussions about their own trustworthiness.

Now, despite the fact that trust audits would likely be crafted by subject-matter experts and conveyed via detailed panel reports, there could never be a single, final, and authoritative trust audit for any given technology company. For one thing, the quality of these assessments would be dependent upon the evidence that an auditing team has access too, as well as upon their skill at interpreting and reasoning through what that information suggests about a particular company’s operations. For another thing, the technology sector is a rapidly evolving space and developers pride themselves on being agile when it comes to adjusting both their products and their business practices. It is quite common to see technology companies shifting operations strategies, committing themselves to new technologies, or even quickly rebranding themselves (Thomas, 2021).

A couple of things follow from this. First, trust audits should be understood and promoted as being provisional in nature. There is nothing surprising about this, since in much the same way that new operations, new opportunities, and new information can affect a company’s valuation, so too can they affect an assessment of its trustworthiness. Second, the provisional nature of trust audits means that these evaluations should not be viewed as a one-time exercise or even a short-term project. Updates, revisions, and replacement audits should be an expected part of the process. Hence, trust auditing should not be thought of or treated as a quick fix but rather embraced as an ongoing endeavor. For a community to do otherwise would be to risk forming and promoting outdated understandings of technology companies’ ethical trustworthiness.

With all the preceding in mind, it seems that there is reason to believe that trust audits would be welcomed; that they would be performed by community appointed teams of trust auditors; and that they would be made available as a regularly updated series of detailed panel reports. Through their dissemination via social and local media, these reports would not only raise public awareness and stimulate debate about the trustworthiness of technology companies. They would also create new opportunities for engagement between members of the public and such businesses. In these ways, trust audits might help to focus public attention and public judgment on the ethical dispositions and competencies of specific technology companies, and thereby prompt such businesses into more fully embracing tech ethics.

## Fitting In

There is one last thing to consider before wrapping up this initial discussion of trust auditing, and that is to think about how this type of intervention would relate to the other key strategies for augmenting tech ethics.

First, it is important to recognize that the use of trust audits would not obviously compete or conflict with the regulatory strategy or the design approach. Trust audits could be implemented alongside these other practices without significantly impinging on their functionality and vice versa. For instance, where the regulatory approach seeks to remedy deficiencies in the practice of tech ethics through legally established norms and sanctions, trust auditing relies on the court of public opinion to pressure technology companies into addressing those same shortcomings. Hence, while the regulatory approach and the practice of trust auditing aim at the same end, the means that they employ are structurally independent of one another. A similar relationship obtains between trust auditing and the design approach. The practice of trust auditing relies on the reactions of community members to create an external incentive for technology companies to participate in tech ethics, whereas the design approach seeks to bypass the issue of motivation entirely. It operates by having designers and engineers work ethical considerations right into the research and development process. So, while these two strategies also aim at the same end, they too use different mechanisms to achieve it.

Second, more than just functioning alongside the regulatory and design strategies, trust auditing is likely to augment these other means of supporting tech ethics. Recall, for example, that one of the regulatory approach’s key shortcomings arises because many parts of the world are so under-resourced that they cannot support a regulatory scheme aimed at the enforcement of tech ethics. The regulatory approach thus leaves many communities without a way to exert control over the technology companies that operate within them. For its part, trust auditing does require some set of community members to have the time, the skills, and the resources to identify, collect, and interpret significant amounts of technical information about technology developers—so there are certain expenses associated with this alternative. However, with both education levels and rates of internet access now soaring in low-income regions, trust audits could plausibly be implemented within most communities on earth (Macharia, 2014). As such, trust auditing can help to fill the void left by the regulatory approach by providing those people living in very low-income settings with a mechanism through which they can influence and engage with the technology companies operating in their communities.

Trust auditing can also help mitigate the regulatory approach’s pacing problem. Remember that the pacing problem arises because, by the time a regulatory scheme has been designed and enacted to deal with the ethical issues surrounding an existing set of technologies, a new set will already be emerging to bedevil that framework. The result is that regulatory systems are constantly trying to play keep-up with an overwhelming wave of ethically challenging products and services being rolled out by technology companies. Trust auditing does not suffer from this pacing problem, since it does not try to directly legislate on the creation, distribution, or employment of individual technologies. Rather, it supports tech ethics by disincentivizing businesses from generating problematic outputs in the first place. Importantly, this means that if trust auditing were successfully implemented, it could actually reduce the strain on the regulatory approach by reducing the number of ethically challenging products and services that regulatory bodies need to keep abreast of.

Trust auditing is also primed to support the design approach. As noted earlier, the design approach is hindered by the fact that technology companies are unlikely to subsidize its practice. That is, they are unlikely to adjust their incentive structures so that designers and engineers are encouraged to flag, modify, or halt projects on ethical grounds. Here again, trust auditing not only avoids such problems, but it may actually help the design approach to overcome them. First, trust auditing does not need to rely on funding from technology companies to be effective. It only needs members of the public to recognize, support, and take the work of trust auditors seriously. When these conditions are met, trust auditing would establish a system of incentives and disincentives that is likely to result in more technology companies aligning their operations with the practice of tech ethics than would otherwise be the case. Second, by leading businesses to more fulsomely embrace tech ethics, trust audits create an opportunity for the design approach to find support that it might otherwise not receive. After all, one good way for a company to demonstrate that is disposed and competent to implement the practice of tech ethics is for it to support its designers and engineers’ attempts to integrate ethical decision-making into the innovation process. Thus, trust audits might actually incentivize companies to embrace the design approach.

## Conclusion

Tech ethics is often put forward as a salve for the public’s mistrust of technology developers. Yet, tech ethics’ remedial potential is severely hampered because the public knows that technology companies are currently incentivized to pursue it half-heartedly. Worse than this, the two main strategies for improving tech ethics suffer from important practical limitations. I have therefore proposed a new way to augment tech ethics: by having members of the public initiate trust audits—community-based evaluations of technology developers’ ethical-trustworthiness. The idea is that technology companies will want to avoid the fallout associated with public judgments of ethical untrustworthiness and will, therefore, seek to participate in the practice of tech ethics more fulsomely. Then, when the public understands that it has the power to target and to pressure self-interested technology companies into becoming better moral actors, its sense that technological innovators are “out of control” should lessen, setting the scene for the regeneration of public trust in technology developers and their products. In order laying out this proposal, I have considered what trust audits might look like in practice and how they would relate to the other strategies for augmenting tech ethics. The result is that trust audits appear to offer a viable new form of public engagement and that they appear able to supplement the other strategies for enhancing the practice of tech ethics. Taken together, this suggests that there are good grounds for continuing to investigate both the idea of trust audits and the promise that they might hold for improving tech ethics and enhancing public trust in the technology industry.

## Footnotes

1 If correct, this would defy Brian Wynne’s claim that when it comes to public participation, “(t)he only thing which one can expect to control, and to take responsibility for, is one’s own trustworthiness” (Wynne 2006).
